# Binding features by relaying modulator group of neurons

**DOI:** 10.1186/1471-2202-12-S1-P7

**Published:** 2011-07-18

**Authors:** Toomas Kirt, Talis Bachmann

**Affiliations:** 1 Laboratory of Cognitive Psychology, University of Tartu, Teatri väljak 3, Tallinn 10143, Estonia

## 

Simultaneous oscillation of neuron groups could provide solution to the binding problem [1] and simultaneously firing neurons could bind together different features of the same object. The corticothalamic system plays a key role in synchronizing the activity of thalamic and cortical neurons [2] and synchronized oscillations have important function in brain activities [3]. It is shown that a relaying neuron group could cause zero time lag synchronization among distant neuron groups [4]. As there are many fields representing different features then they compete with each other to become dominant; thus, Jörg Lücke [5] proposed that there might be multiple bifurcation points where the network tends to move towards a certain stable state. Such selection is achieved by the decision making processes [6], where a common group of inhibitory neurons inhibits activity of the to-be-loosing excitatory neuron group. Based on this we have built up an experimental setup where we used two pairs of exclusive features from two dimensions and tried to bind together one from each dimension as a unified object and cause activity of the higher level group of neurons.

**Figure 1:**
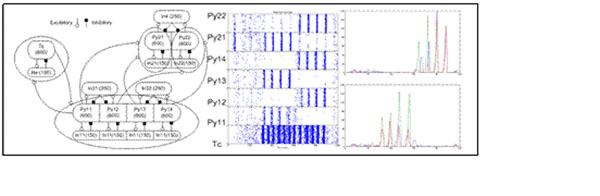
A) Network structure. B) Raster plot of spikes of excitatory neurons. C) Averaged firing histogram of subpopulations. Upper figure Py12 and Py14 subpopulations and their common firing (blue, green and red respectively) and lower figure Py11 and Py13 (the same color scheme).

In our experiments the Brian simulator [7] featuring a leaky integrate-and-fire neuron model is used and three main groups of excitatory neurons are formed. The first group is the modulator group (see Figure 1A Tc) which synchronizes activity of the next feature group (Py1) and the latter sends synaptic effects to the higher consolidating group (Py2). All the excitatory neurons are connected to a local inhibitory group (Re, In1, In2) and the feature neurons and consolidating neurons get pairwise additional inhibitory input (In3, In4). All the neurons receive Poissonian background input noise that causes averaged network activity in the frequency of 1 Hz. The neurons from modulator and feature groups get additional Poissonian input in a certain time frame and, during the simulations, activity switches from one pair of features to the other. As a result we could see the well synchronized activity of feature neurons could cause oscillating activity of neurons in the higher group (Figure 1).

There might be some number of basic mechanisms that are responsible for processing and forwarding information in the brain, including a variety of synchronizing mechanisms. In this experiment we have shown how synchronized oscillations of neurons and decision making processes can be used for binding and forwarding information between the neuron groups.
